# University of Michigan

**Published:** 1861-07

**Authors:** 


UNIVERSITY OF MICHIGAN.—Dr. S. G. Armor has accepted the Professorship of the Institutes of Medicine and Materia Medica in the University of Michigan, an institution which is now attracting large classes, that of last winter having numbered 242.

There are some features in regard to the plan of instruction in this school which might be profitably imitated by other similar institutions. Thus, it has a session of six months, and four lectures only are delivered a day; an arrangement which affords the student ample time for dissections, careful reading, and chemical manipulations. The professors are paid by the State, and no pupil is permitted to leave before the close of the session, his tickets being withheld up to that period.

